# Upregulated Angiogenesis Is Incompetent to Rescue Dilated Cardiomyopathy Phenotype in Mice

**DOI:** 10.3390/cells10040771

**Published:** 2021-03-31

**Authors:** Mohammed Arif, Perwez Alam, Rafeeq PH Ahmed, Raghav Pandey, Hafeez M Faridi, Sakthivel Sadayappan

**Affiliations:** 1 Heart, Lung and Vascular Institute, Department of Internal Medicine, Division of Cardiovascular Health and Disease, College of Medicine, University of Cincinnati, Cincinnati, OH 45267, USA; SADAYASL@ucmail.uc.edu; 2 Department of Pathology and Laboratory Medicine, College of Medicine, University of Cincinnati, Cincinnati, OH 45267, USA; alampz@ucmail.uc.edu (P.A.); rafeeqhabeeb@gmail.com (R.P.H.A.); raghav.pandey365@gmail.com (R.P.); 3 Department of Pharmaceutical Sciences, College of Pharmacy, Chicago State University, Chicago, IL 60628, USA; hfaridi@csu.edu

**Keywords:** dilated cardiomyopathy, angiogenesis, MicroRNA-210

## Abstract

Dilated cardiomyopathy (DCM) is characterized by pathologic cardiac remodeling resulting in chambers enlargement and impaired heart contractility. Previous reports and our in-silico analysis support the association of DCM phenotype and impaired tissue angiogenesis. Here, we explored whether the modulation in cardiac angiogenesis partly intervenes or rescues the DCM phenotype in mice. Here, a DCM mouse model [α-tropomyosin 54 (α-TM54) mutant] was crossbred with microRNA-210 transgenic mice (210-TG) to develop microRNA-210 (miR-210) overexpressing α-TM54 mutant mice (TMx210). Contrary to wild-type (WT) and 210-TG mice, a significant increase in heart weight to body weight ratio in aged mixed-gender TMx210 and DCM mice was recorded. Histopathological analysis revealed signs of pathological cardiac remodeling such as myocardial disarray, myofibrillar loss, and interstitial fibrosis in DCM and TMx210 mice. Contrary to WT and DCM, a significant increase in angiogenic potential was observed in TMx210 and 210-TG mice hearts which is reflected by higher blood vessel density and upregulated proangiogenic vascular endothelial growth factor-A. The echocardiographic assessment showed comparable cardiac dysfunction in DCM and TMx210 mice as compared to WT and 210-TG. Overall, the present study concludes that miR-210 mediated upregulated angiogenesis is not sufficient to rescue the DCM phenotype in mice.

## 1. Introduction

Cardiovascular diseases (CVD) resulting in heart failure are the leading cause of mortality worldwide. Currently, CVD poses an economic burden of more than $215 billion annually in the United States alone which is projected to rise to $1208 billion each year by 2030 [1]. Dilated cardiomyopathy (DCM) is one of the most prevalent non-ischemic cardiomyopathies, originating due to multiple causes including mutations in cardiac sarcomeric protein-encoding genes [2]. Moreover, DCM is the most common lethal form of pediatric cardiomyopathy; often without any known cause [3]. The hallmark of DCM comprises enlargement of ventricular chambers with successive wall thinning due to cardiomyocyte death followed by fibrosis leading to myocardial dysfunction. This results in systolic pump impairment and arrhythmia leading to congestive heart failure. Global research trials are in progress to reverse such abnormal cardiac remodeling and restore normal heart function post-DCM, although none of these have transitioned into routine clinical use. Expensive and aggressive surgeries like cardiac transplants remain the only viable option to date. Thus, considering the high cost, a limited number of donors, and an ever-increasing demand for organ transplants, there is an immediate and essential need for the development of the least invasive and cost-effective ideal therapy for DCM.

Angiogenesis plays key roles during tissue development and homeostasis and impaired angiogenesis is closely related to several diseases, including CVD [4]. Preliminary studies in animal models and human patients suggest the key roles played by tissue angiogenesis in pathologic cardiac remodeling during ischemic injury [5] and DCM [6,7,8,9]. Hence, angiogenesis intervention strategies have been explored clinically for DCM therapeutics [10]. Studies with transplant patients with DCM have reported a significant decrease in cardiac angiogenic factors [such as vascular endothelial growth factor A (VEGF-A) and vascular endothelial growth factor receptor 1 (VEGFR1)] expression and lower capillary density [11], suggesting downregulation of cardiac angiogenesis during DCM phenotype. Promoting tissue angiogenesis during DCM has been shown to have an anti-fibrotic and cardioprotective effect [12,13,14]. Overall, depending on this information, we hypothesized that augmenting angiogenesis in the DCM heart could be a possible novel strategy that could be translated into promising therapy. Earlier reports have shown that microRNAs, a group of small non-coding RNAs, could play key regulatory roles during various physiological and pathological processes, including angiogenesis [15,16]. Likewise, our previous study has revealed the beneficial effects of microRNA-210 (miR-210) mediated upregulated angiogenesis during cardiac injury [16]. Thus, based on reported beneficial effects of miR-210 via promoting cardiac tissue angiogenesis, we raised the question, could miR-210 mediated increase in cardiac angiogenesis modulate the DCM phenotype? To test this notion, we overexpressed miR-210 in DCM mice to improve cardiac angiogenesis to reverse pathologic cardiac remodeling. However, miR-210 mediated upregulated angiogenesis was found to be insufficient to rescue DCM phenotype and heart function in miR-210 overexpressing DCM mice. 

## 2. Materials and Methods

Chemicals and reagents were purchased from Sigma-Aldrich, Saint Louis, MO, USA unless otherwise mentioned. Antibodies used in the current study were obtained from Sigma-Aldrich, MO, USA (Rabbit anti-GAPDH, Cat. G9545; mouse anti-α-smooth muscle actin, Cat. A5228); Santa Cruz Biotechnology, TX, USA (Rabbit anti-cardiac troponin-I, Cat. sc-15368; rabbit anti-VEGF-A, Cat. sc-152); GE Healthcare, IL, USA (ECL anti-rabbit-HRP, Cat. NA9340V); Life Technologies, Carlsbad, CA, USA (Goat anti-mouse Alexa Fluor 488, Cat. A11029; goat anti-mouse Alexa Fluor 594, Cat. A11005; goat anti-rabbit Alexa Fluor 488, Cat. A11008; goat anti-rabbit Alexa Fluor 594, Cat. A11037); VWF (von Willebrand factor) (Dako, Carpinteria, CA, USA, Cat. GA527).

Animals were maintained at Laboratory Animal Medical Services (LAMS), University of Cincinnati, USA. The animals were fed ad libitum standard feed and were utilized per the protocol approved by the Institutional Animal Care and Use Committee (IACUC).

### 2.1. Dilated Cardiomyopathy (DCM), MicroRNA-210 (MiR-210) Transgenic, and α-TM54 Mutant (TMx210) Mice Models

As reported earlier [17], here we used a DCM mice model having a point mutation within α-tropomyosin at Glu54Lys (Figure S1), which is similar to the human counterpart. These DCM mice were produced in the laboratory of Dr. David F. Wieczorek who also donated these mice to us; they were further maintained at the University of Cincinnati animal facility. Also, miR-210 overexpressing (global) transgenic mice (210-TG) were generated at the University of Cincinnati Transgenic core using the C57BL/6 mice strain as mentioned elsewhere [16] (Figure S2). Similarly, DCM (white coat) and 210xTG (black coat) mice were crossed to get TMx210 (grey coat) mice. 

### 2.2. Mice Genotyping

Tail tissues from all weaned mice were digested in 100 µL digestion buffer (10 µL 10× polymerase chain reaction (PCR) buffer, 25 mM MgCl_2_, 10 µL 10mg/mL proteinase K, 61 µL nuclease-free water, 4.5 µL 10% Tween 20, 4.5 µL 10% NP-40) for 5 h at 60 °C. Samples were boiled for 10 min followed by a 5-s vortex and centrifuged at 10,000 RPM for 5 min. Supernatant was used to set up the Multiplex PCR using the PCR reaction mix and custom-designed primers (5 µL genomic DNA sample, 5 µL 10× PCR buffer, 10 µL Q-solution, 1 µL test gene and Glyceraldehyde 3-phosphate dehydrogenase (GAPDH) forward and reverse primers, 1 µL 10 mM dNTP, 22.5 µL water, 0.5 µL Taq Polymerase) (Table S2). PCR conditions were set as 4 min 94 °C, 34 cycles of 30 s 94 °C, 30 s 55 °C and 30 s 70 °C, and 10 min 72 °C followed by 25 min 4 °C. PCR product was resolved via 1.5% agarose gel electrophoresis and DNA bands were analyzed under the ultraviolet (UV) gel doc system (BioRad).

### 2.3. Histological Examination and Immunohistochemistry

Before isolation, hearts were arrested in the diastolic phase with 1 M KCl and perfused and fixed in 10% buffered formalin solution for 48 h at room temperature (RT). Tissue samples were fixed in paraffin blocks and 5 micron thick sections were cut using a microtome. Formalin-fixed tissue slides were de-paraffinized via incubation at 70 °C for 30 min followed by immediately dissolving paraffin in Xylene, and re-hydrating through sequential 5 min incubations in graded ethanol (100%, 90%, and 80%) and water. Antigen retrieval was performed via heating the slides in a pressure cooker in 0.1 M citrate buffer (pH-6.0) for 3 min followed by cooling in the same buffer for 45 min. Tissue sections were 3× washed with PBS (phosphate buffered saline, pH-7.3), blocked with 3% BSA (bovine serum albumin) for 1 hr. Then they were washed with PBS and slides were incubated with the primary antibody in 3% BSA against analyzing antigen for 1–3 h at 37 °C followed by incubation with appropriate secondary antibody linked with Alexa fluor for 1 h at 37 °C. Slides were incubated with DAPI for 10 min, washed with PBS twice, and mounted with Fluoromount-G (Southern Biotech, AL, USA). Slides were examined using a confocal microscope (Olympus). Cell area/size was defined by the staining of cardiac tissue with wheat germ agglutinin (WGA) (Invitrogen, CA, USA). To analyze angiogenesis, blood vessels were stained using anti-smooth muscle actin and anti-troponin I antibodies.

### 2.4. Echocardiography 

Indices of left ventricular function and chamber dimensions during systole and diastole were examined via transthoracic echocardiography (Echo-), performed on one-, three-, and six-month-old mice using a Vevo 2100 Imaging digital ultrasound system (VisualSonics) with a 22–55 MHz (MS550D) transducer probe. Before echocardiography, mice were anesthetized with oxygen mixed with isoflurane (2–2.5%). Mice chest hairs were shaved and then it was placed on a warm platform in the supine position. Data acquisition was initiated with the parasternal cardiac long axis view followed by a transition to the short axis view, at the level of mid-papillary muscles. Measurements were obtained from long-axis M-mode images, and graphically represented as % LVEF (left ventricular ejection fraction), % LVFS (left ventricular fraction shortening), LVID;d (left ventricular internal diameter end diastole) (mm), LVID;s (left ventricular internal diameter end-systole) (mm), LVPW;d (left ventricular posterior wall diastole) (mm), and LVPW;s (left ventricular posterior wall systole) (mm).

### 2.5. In Silico Analysis

DCM-associated genes were pulled out using the DisGeNET database (http://www.disgenet.org/search, accessed on 20 August 2020). These genes were further evaluated using Cytoscape 3.6.1 (ClueGo plugin) for signaling pathway analysis. Common genes related to DCM and angiogenesis were filtered and illustrated in the form of gene networks for Gene Ontology (GO): biological process, Kyoto Encyclopedia of Genes and Genomes (KEGG) pathways, and Reactome pathways.

### 2.6. Tube Formation Assay: Adult Rat Cardiomyocyte (ARCM) Isolation, and Cell Culture

Primary cultures of ventricular cardiomyocytes were obtained from 12 weeks old Fisher rats. Briefly, rats were euthanized, hearts were dissected and perfused using solution A (118 mM NaCl, 4.8 mM KCl, 25 mM HEPES, 1.25 mM MgSO_4_, 1.25 mM K_2_HPO_4_, 10 mM glucose, 4.95 mM taurine, 9.89 mM 2,3-butanedione monoxime pH-7.35). Hearts were then adjusted on the Langendorff system and digested using digestion buffer (solution A with 0.1% BSA, 0.05 mM CaCl_2_, 0.07% collagenase type II, 0.02% hyaluronidase type I). Then ventricular tissue was taken and minced in digestion buffer. The cell suspension was filtered using a 100-micron cell strainer (BD Biosciences, CA, USA) and the filtrate was centrifuged at 300 rpm for 3 min at RT. The cell pellet was suspended in solution B (solution A with 1% BSA, 0.1 mM CaCl_2_) and cells were allowed to settle under gravity. The cell pellet was resuspended in Dulbecco’s modified Eagle medium (DMEM)/high glucose media (GE Healthcare, UK) containing 10% fetal bovine serum (FBS) (Fisher Scientific, MA) and 1% Penicillin/Streptomycin (Fisher Scientific, MA, USA) and seeded on culture plates. One day post-seeding, cells were transfected with *Caenorhabditis elegans* specific miR, cel-miR-67 (50 nM) (Dharmacon, CO, USA) (negative control), and rno-miR-210 mimic (50 nM) (Dharmacon, CO, USA), using Lipofectamine RNAiMAX reagent (Life Technologies, CA, USA). Post 24 h following transfection, media was removed and fresh culture media was added. Cells were cultured for one-week post-transfection and conditioned media was collected. In another parallel setup, seeded HUVEC (human umbilical vein endothelial cells) were cultured for 6–8 h using 50% conditioned media +50% endothelial growth media (Corning, NY, USA) containing 2.5% fetal bovine serum. In a positive control setup, VEGF (Vascular endothelial growth factor; 750 ng/mL medium) and bFGF (basic fibroblast growth factor; 500 ng/mL medium) were added in the HUVEC culture medium. Then, tube formation analysis was performed by evaluating the total tube length, and total branching points. 

### 2.7. RNA Extraction and Quantitative Polymerase Chain Reaction (qPCR)

Total RNA (including miRNAs) was extracted from mice hearts using the PAXgene kit (Qiagen, Cat. 763134) following the manufacturer protocol. The RNA concentration was estimated using nanodrop and purity was checked by A260/280 ratio >1.8. Purified RNA was further used for cDNA synthesis using miScript II RT kit (Qiagen, Cat. 218161) followed by qPCR for miR-210 using custom-designed primers (Table S3), miScript Primer assay kit (Qiagen, Cat. 218300), and miScript SYBER Green PCR kit (Qiagen, Cat. 218075) on a Bio-Rad Real-Time PCR Detection System. Results were normalized with an internal control (such as miR-U6) and analyzed via the comparative C_t_ method. 

### 2.8. Aortic Ring Assay

To study angiogenic potential in mice groups, an aortic ring assay was performed according to a published protocol [18] with minor changes. In short, six-month-old mice were euthanized, and aorta, located along with the spine, were dissected, cleaned in Opti-MEM media, and cut into rings of 1 mm width. Rings were serum-starved overnight in Opti-MEM at 37 °C and 5% CO_2_ followed by embedding in Matrigel matrix (Sigma, MO, USA). Rings were fed with 199 media (Sigma-Aldrich, MO, USA) containing 2.5% FBS, 1% Penicillin/Streptomycin. Post one week, rings were fixed with 4% paraformaldehyde in PBS for 20 min followed by 3× washes with PBS. Under bright field imaging, neovascularization area and number of microvessel sprouts were counted in each aortic ring.

### 2.9. Tissue Lysate Preparation, Sodium Dodecyl Sulfate (SDS) Gel Electrophoresis, and Western Blotting

Mice hearts were collected, perfused with PBS, and homogenized in Laemmli buffer with β-mercaptoethanol followed by immediate boiling in a hot water bath for 20 min. Subsequently, the lysate was centrifuged at 12,000× *g* for 30 min at 4 °C and the supernatant was stored at −80 °C until use. Protein content was estimated using the Pierce 660 nm reagent kit (Thermo Scientific, Waltham, MA, USA). Linear slab gel electrophoresis under the denaturing condition (in the presence of 0.1% sodium dodecyl sulfate (SDS)) was performed. Protein samples were electrophoresed using 10–12% polyacrylamide gel (Invitrogen, CA, USA) and running buffer (containing 0.025 M Tris-base, 0.192 M glycine pH 8.3, and 0.1% SDS) in Novex apparatus (Life Technologies, CA, USA). The standard molecular weight marker was electrophoresed alongside to observe the subunit size of the analyzed protein. Electrophoresed protein samples were electroblotted onto PVDF membrane by applying 0.8 mA/h current in a wet transfer unit (Bio-Rad, Hercules, CA, USA), and were detected using rabbit/mouse primary antibody and anti-rabbit/mouse conjugated horseradish peroxidase (HRP) second antibody. Blots were incubated for 2–3 min with SuperSignal W. Femto max sensitivity (Thermo Fisher, MA, USA) enhanced chemiluminescence (ECL) substrate. Blots were kept in a cassette; a photographic film was exposed to it and developed to visualize the bands for analyte proteins.

### 2.10. Statistical Analysis

Student’s *t*-test was used to identify significant differences in quantitative values between the two test groups. For more than two groups one way analysis of variance (ANOVA) was performed. All studies or experiments were performed independently at least three times (*n* = 3), to achieve statistical power and *t*-test significance (where * *p* < 0.05; ** *p* < 0.01; *** *p* < 0.001, two-tailed distribution, paired). Wherever needed, the graphical approach was done for further analysis and presentation of data by using the GraphPad Prism 7.04 program.

## 3. Results

### 3.1. DCM Mice Model

**Figure 1:**
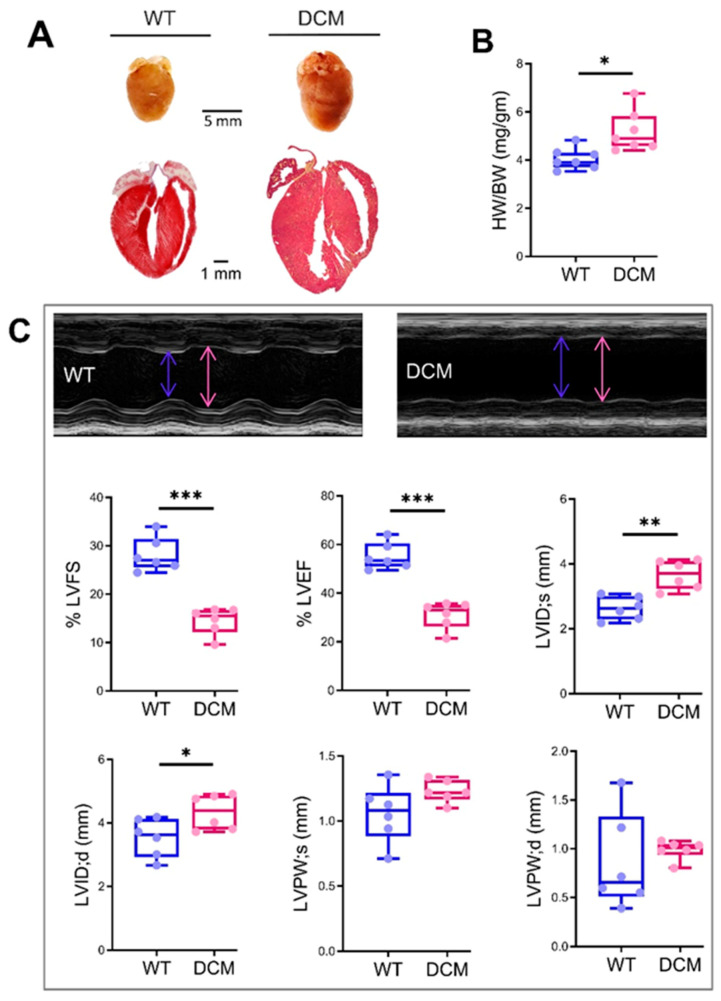
α-Tropomyosin 54 mutant mouse is a model for the dilated cardiomyopathy (DCM) phenotype. (**A**,**B**) Masson Trichrome stained histological sections, and heart weight to body weight (HW/BW) ratio of three months old mixed-gender wild-type (WT) and α-Tropomyosin 54 (α-TM54) mutant mice hearts (DCM) showing the bigger cardiac size in the latter group. (**C**) Echocardiography analysis showing elevated LVEF, LVFS, LVID;d, LVID;s, LVPW;d and LVPW;s in DCM as compared to WT. The sample size (N) is indicated by respective color dots over the box and whiskers plots. Here * *p* < 0.05; ** *p* < 0.01; *** *p* < 0.001. LVEF: Left ventricular ejection fraction; LVFS: Left ventricular fraction shortening; LVID;d: Left ventricular internal diameter end diastole; LVID;s: Left ventricular internal diameter end-systole), and LVPW;d: Left ventricular posterior wall diastole; LVPW;s: Left ventricular posterior wall systole.

As reported earlier [17], α-Tropomyosin 54 (α-TM54) mutant transgenic mice (represented as DCM group) were presented here as a DCM model. By contrast with wild-type (WT), three-month-old α-TM54 mutant mice showed a comparatively bigger size heart (Figure 1A), ~30% elevation in heart weight to body weight ratio (HW/BW) (Figure 1B), ~55% decrease in %LVEF (left ventricular ejection fraction), ~50% decrease in %LVFS (left ventricular fraction shortening), ~20% increase in LVID;d (left ventricular internal diameter end diastole), and ~35% increase in LVID;s (left ventricular internal diameter end-systole) (Figure 1C). However, there were no significant changes observed in LVPW;d and LVPW;s. Overall, these observations suggest pathologic cardiac remodeling, ventricular chamber dilation, and cardiac dysfunction, thus representing the hallmarks of a DCM phenotype. Also, to analyze whether these DCM mice died due to heart failure with aging, we aged these mice until they were six months old and we observed a relatively more severe form of ventricular dilation (as indicated by a significant increase in LVID;d and LVID;s), wall thinning (as indicated by a significant decrease in LVPW;d and LVPW;s), and cardiac dysfunction in all of the DCM mice, however, all the animals survived during aging (Table S1 and Figure S3).

### 3.2. DCM Phenotype Involves Angiogenic Signaling

**Figure 2:**
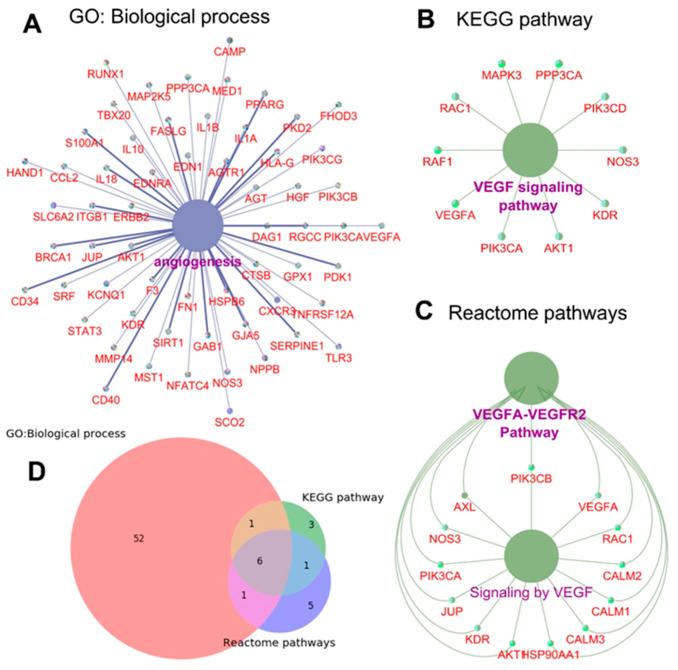
DCM phenotype has a close relationship with tissue angiogenesis. (**A**–**C**), In silico analysis using DisGeNET database and Cytoscape 3.6.1 (ClueGo plugin) tool indicating GO: biological process (**A**), KEGG (**B**), and Reactome pathways (**C**) showing proangiogenic genes or angiogenesis pathway involved in DCM phenotype. (**D**), Venn diagram illustrating correlation analysis showing the number of shared genes from different databases. GO: Gene Ontology; KEGG: Kyoto encyclopedia of genes and genomes.

To explore the relationship between DCM phenotype and angiogenesis, as reflected by earlier reports [6,9], we performed bioinformatics analysis using different in silico tools (such as the DisGeNET database and Cytoscape 3.6.1 with ClueGo plugin) which suggest that DCM phenotype involves several proangiogenic genes and related signaling pathways (Figure 2A–D). This information indicates a direct association between DCM development/pathogenesis and tissue angiogenesis.

### 3.3. MiR-210 Promotes Cardiac Tissue Angiogenesis

**Figure 3:**
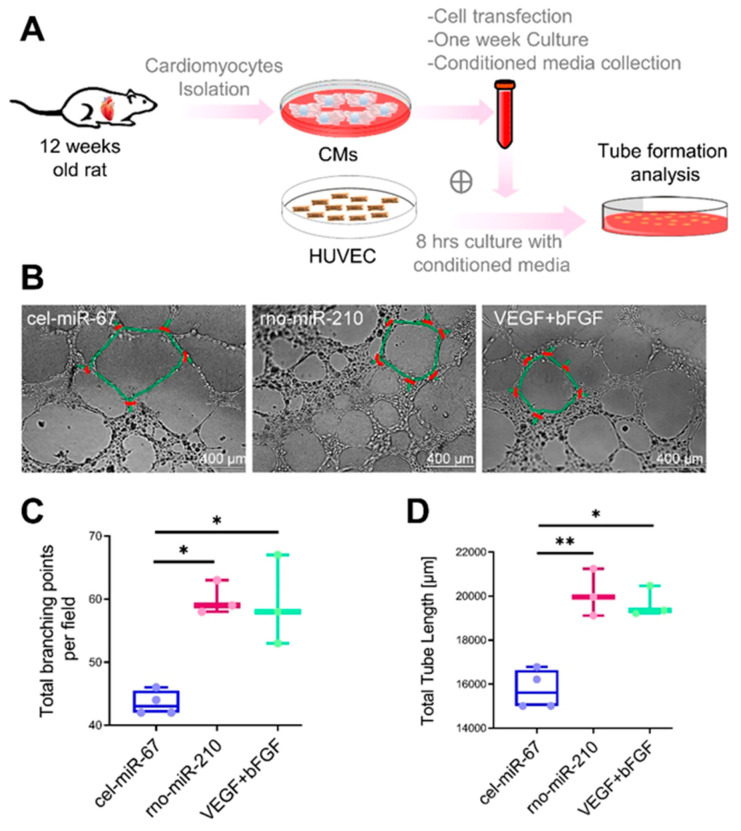
MicroRNA-210 (MiR-210) induces angiogenesis under in vitro conditions. (**A**), Experimental design workflow illustrating cardiomyocytes (CMs) isolation from adult Fisher rat, transfection with cel-miR-67 (control), and rno-miR-210 mimic followed by one week culture under controlled conditions. An equal amount of CMs culture conditioned media was used along with endothelial growth media to culture HUVEC for 6–8 h followed by tube formation analysis. VEGF (750 ng/mL medium) and bFGF (500 ng/mL medium) were added in the culture medium to culture HUVEC as a positive control setup (VEGF + bFGF). (**B**), Representative bright-field images of HUVEC, post 8 h culture in conditioned media showing tubes (indicated by green outlines) with different branching points (indicated by red points). (**C**,**D**), Box, and whiskers plots illustrating quantification of the tube length (**C**), and branching points (**D**) showing a significant increase in tube formation and thus upregulated angiogenic potential in miR-210 and VEGF+bFGF groups as compared to cel-miR-67 control. Here * *p* < 0.05; ** *p* < 0.01. HUVEC: Human umbilical vein endothelial cell; VEGF: Vascular endothelial growth factor; bFGF: Basic fibroblast growth factor.

As reported earlier, miR-210 can induce angiogenesis in mice hearts following injury [16]. Thus, we analyzed the angiogenic potential of miR-210 using the in vitro system. Likewise, adult rat cardiomyocytes were transfected with rno-miR-210 and culture for one week. At one-week post-transfection, conditioned media (containing all the secreted proangiogenic factors) was added to the culture of HUVEC and incubate for 6–8 h followed by tube formation analysis (Figure 3A). Quantification of the angiogenesis indicators suggested a ~25% and ~40% increase in total tube length and total branching points respectively in the miR-210 group as compared to cel-miR-67 control, indicating a higher angiogenic potential in the former group (Figure 3B–D).

### 3.4. TMx210 Mice Show Comparable Cardiac Phenotype to DCM Hearts

**Figure 4:**
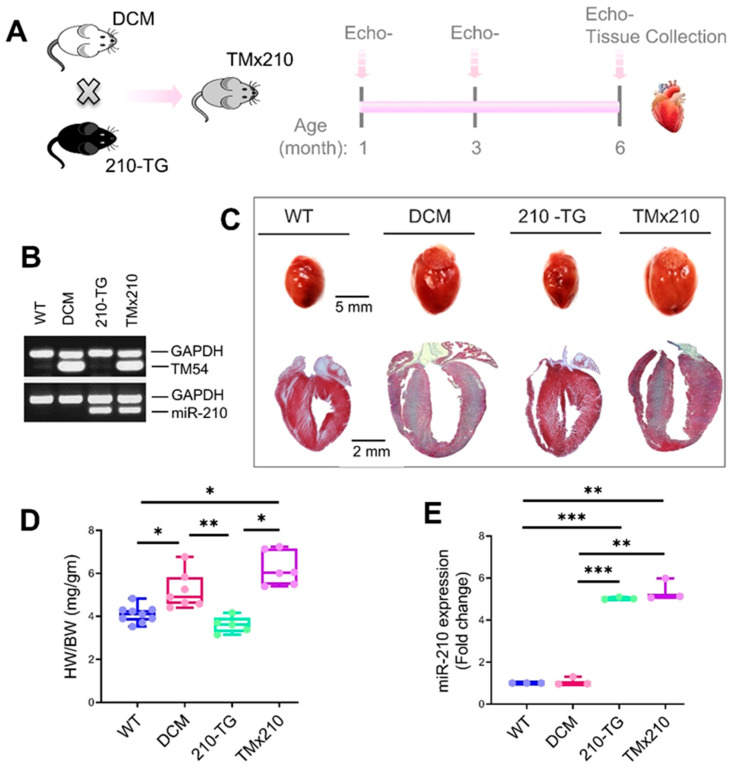
Transgenic animal models. (**A**) α-tropomyosin 54 (α-TM54) mutant mice (DCM) were crossed with miR-210 overexpressing transgenic mice (210-TG) to achieve miR-210 overexpressing α-TM54 mutant mice (TMx210). Echocardiography was performed at three and six months of age following the killing of all mice to dissect the heart tissue for all molecular analyses. (**B)** Genotyping of mice tail genomic DNA via multiplex polymerase chain reaction (PCR) using custom-designed primers (Table S2) showing wild-type (WT; lane 1), DCM (lane 2), miR-210 transgenic (210-TG; lane 3), and TMx210 (lane 4). GAPDH was used as an internal control. (**C**) Representative images of heart tissues and Masson Trichrome stained heart sections following six months age, illustrating cardiac size difference in different groups. (**D**) Heart weight to body weight ratio (HW/BW) in different groups showing the comparison of heart size. (**E**), Quantification of cardiac miR-210 expression by qPCR showing miR-210 overexpression in 210-TG and TMx210 hearts. Here * *p* < 0.05; ** *p* < 0.01; *** *p* < 0.001. GAPDH: Glyceraldehyde 3-phosphate dehydrogenase.

To analyze the effect of miR-210 induced angiogenesis in DCM hearts, we crossbred miR-210 overexpressing transgenic mice (210-TG) [16] (Figure S2), with α-tropomyosin 54 (α-TM54) mutant (DCM) mice [17] to achieve miR-210 overexpressing α-TM54 mutant mice (TMx210) (Figure 4A). Genotyping using tail tissue confirmed the expression of transgenes miR-210 and mutant α-TM54 in TMx210 mice as compared to WT (wild-type) (Figure 4B). All mice in each group survived during aging, and we did not document any death in any group. At six months of age, assessment of Masson Trichrome stained heart sections (Figure 4C) and HW/BW ratio (Figure 4D) in different groups suggested the significant increase in cardiac size in DCM and TMx210 mice as compared to WT and 210-TG. As the bigger heart size may also be due to cardiac hypertrophy, thus, the cardiomyocyte size (via immunohistochemistry), and cardiac chamber dilation (via Echo- analysis) were also analyzed in later sections, which would confirm the DCM phenotype. Also, qPCR-based quantification for cardiac miR-210 expression demonstrated a ~5 fold increase in miR-210 levels in 210-TG and TMx210 groups as compared to WT and DCM mice (Figure 4E).

**Figure 5:**
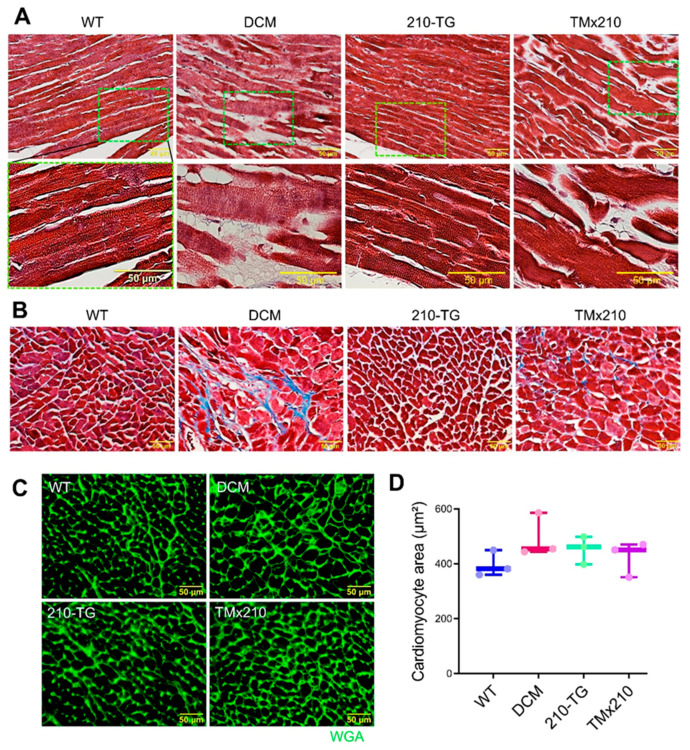
TMx210 mice undergo pathologic cardiac remodeling. (**A**,**B**) Histopathological high magnification images of Masson Trichrome stained heart sections showing myofibrillar loss (as indicated by abnormal myofibrils) (**A**), and interstitial fibrosis (as indicated by blue-colored collagen deposits) (**B**) in six months old DCM and TMx210 mice suggesting the pathologic cardiac remodeling. (**C**) Representative immunostained heart sections at six months age, illustrating WGA (green) stained cardiac cells. (**D**) Quantification of cell cross-sectional area using WGA stained cardiac tissue sections presenting the comparable cell size in different groups. WGA: Wheat germ agglutinin.

Likewise, histopathological analysis of high magnification images of Masson Trichrome stained heart sections showed poorly aligned abnormal myofibrils (suggesting myocardial disarray and myofibrillar loss) (Figure 5A) as well as blue colored patches representing collagen deposits due to interstitial fibrosis (Figure 5B) in DCM and TMx210 mice suggesting the pathologic cardiac remodeling. Although we did not quantify the fibrosis level, via comparing multiple high magnification images, we observed a higher amount of interstitial collagen deposition in DCM hearts sections as compared to TMx210. Also, immunohistochemistry and assessment of cell area revealed a comparable cardiomyocyte size in all the groups; however, there was an uptrend for bigger cardiomyocyte size in DCM hearts (Figure 5C,D). These observations reflect the absence of cardiac cell hypertrophy in all groups.

### 3.5. TMx210 Mice Show Upregulated Angiogenic Potential

**Figure 6:**
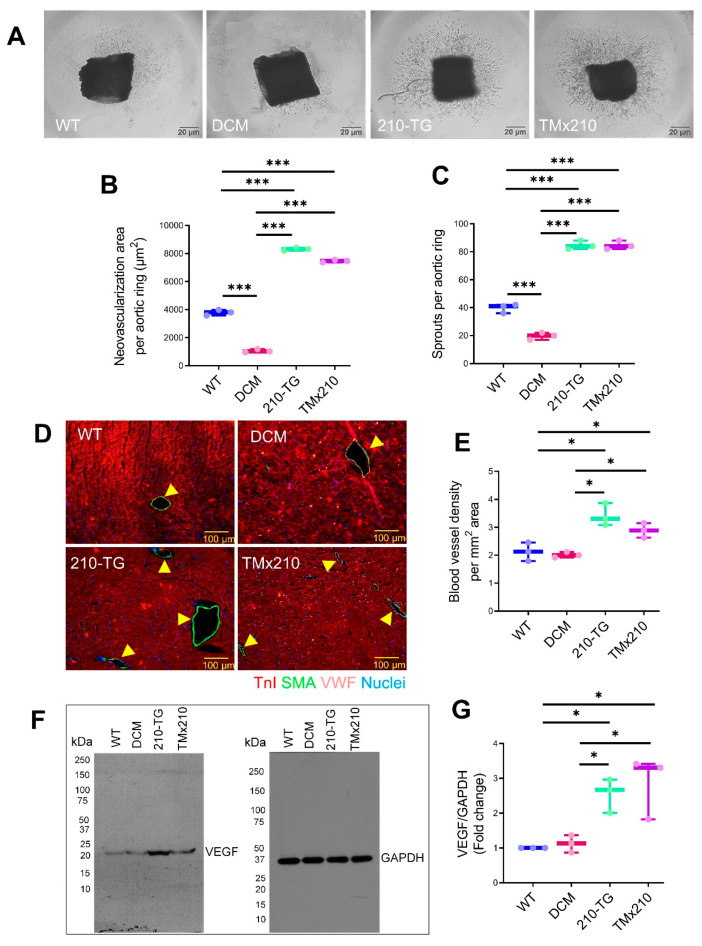
MiR-210 overexpression promotes angiogenesis in TMx210 mice heart. (**A**) Representative bright-field images for aortic ring assay performed in six-month-old mice, illustrating aortic neovascularization, indicated by microvessels sprouting in different groups. (**B**,**C**), Box, and whiskers plots depicting quantification of neovascularization area around each aorta (**B**), and several aortic sprouts per aortic ring (**C**) show a significant increase in neovascularization in 210-TG and TMx210 groups. (**D**) Immunostained mice heart sections, showing blood vessels (arrows) illustrated via multilayered smooth muscle actin (green) and von Willebrand factor (VWF, pinkish-red) positive cells, and troponin I stained cardiac cells (red). (**E**) Quantitative analysis of blood vessel density in whole heart sections, indicating a significant increase in the number of blood vessels in 210-TG and TMx210 hearts. (**F**,**G**), Western blot (**F**), and densitometric analysis (**G**) showing significant upregulation of angiogenic factor VEGF-A in 210-TG and TMx210 hearts. GAPDH was used as a loading control. Here * *p* < 0.05; *** *p* < 0.001. VEGF-A: Vascular endothelial growth factor A; GAPDH: Glyceraldehyde 3-phosphate dehydrogenase.

To compare the angiogenic potential in different groups, an aortic ring assay was performed (at six months of age) that showed ~two-fold increase in neovascularization area, and aortic microvessel sprout formation in 210-TG and TMx210 groups as compared to WT and DCM (Figure 6A–C), suggesting significant upregulation of angiogenic potential in 210-TG and TMx210 mice.

Similarly, in a parallel experiment, immunohistochemistry and quantification of blood vessels in all groups showed a ~50% increase in blood vessel density in 210-TG and TMx210, indicating the upregulation of tissue angiogenesis (Figure 6D,E). As further supporting evidence, Western blots densitometric analysis illustrated ~2.5 folds upregulated expression of angiogenic factor VEGF-A in 210-TG and TMx210 hearts as compared to WT and DCM hearts (Figure 6F,G). Overall, these observations suggest the upregulated angiogenic potential in 210-TG and TMx210 mice.

### 3.6. Upregulated Angiogenesis in TMx210 Mice Is Insufficient to Rescue the Cardiac Function

**Figure 7:**
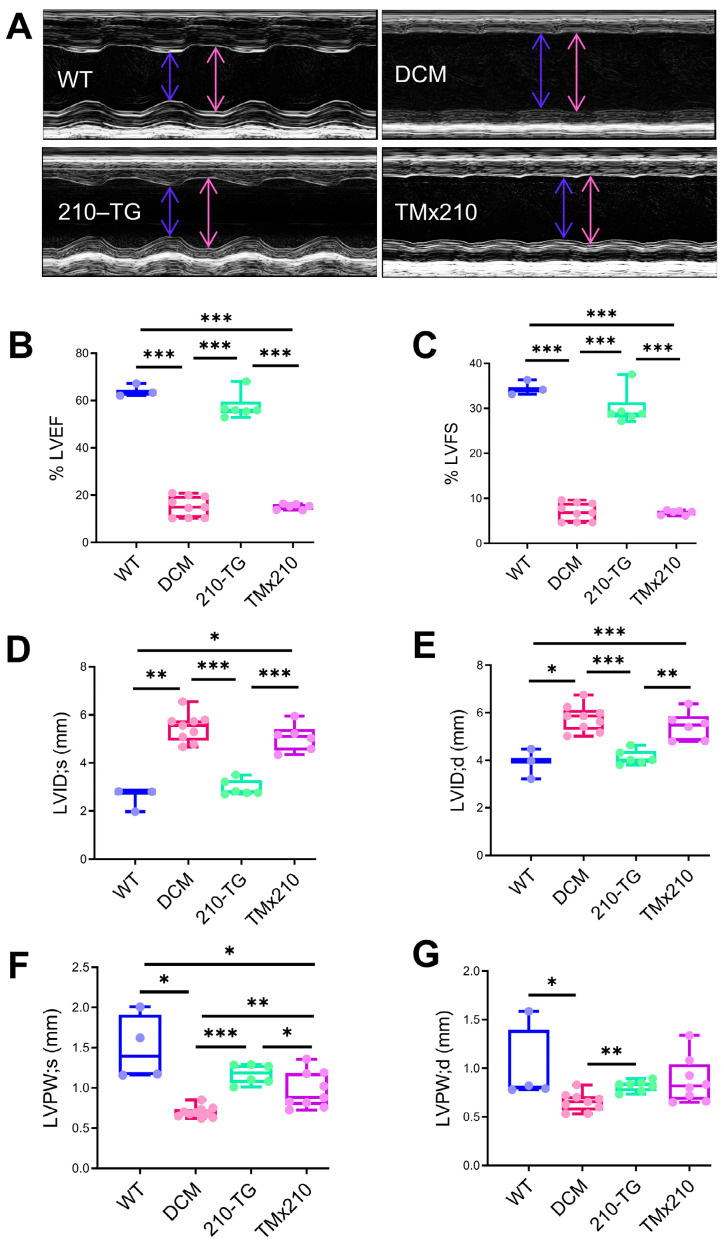
Upregulated angiogenesis does not rescue heart function in TMx210 mice. (**A**) Representative images for echocardiography analysis at six months age in different groups. (**B**–**G**) Echocardiographic assessment, and box and whiskers plots illustrating quantification of heart function in each group showing comparable LVEF, LVFS, LVID;d, LVID;s, LVPW;d and LVPW;s in DCM and TMx210 as compared to WT and 210-TG hearts. Here * *p* < 0.05; ** *p* < 0.01; *** *p* < 0.001. LVEF: Left ventricular ejection fraction; LVFS: Left ventricular fraction shortening; LVID;d: Left ventricular internal diameter end diastole; LVID;s: Left ventricular internal diameter end-systole), and LVPW;d: Left ventricular posterior wall diastole; LVPW;s: Left ventricular posterior wall systole.

To evaluate and compare the effect of upregulated angiogenesis in TMx210 hearts, echocardiography was performed at six months of age (Figure 7A). Analysis of heart function in each group, showed a similar ~four fold decrease in %LVEF, ~four folds decrease in %LVFS, ~40% increase in LVID;d, and ~two-fold increase in LVID;s, in DCM and TMx210 groups as compared to WT and 210-TG hearts (Figure 7B–E), Overall, these data suggest no restoration of heart function in TMx210 mice even having an elevated angiogenic potential. However, LVPW;d (left ventricular posterior wall diastole), and LVPW;s (left ventricular posterior wall systole), both were increased in TMx210 (Figure 7F,G), suggesting a decrease in LV wall thinning compared to DCM hearts.

## 4. Discussion

DCM is one of the most common causes of end-stage heart failure with limited treatment options.

Pathologic cardiac remodeling, consisting of abnormal ventricular geometry, abnormal cardiomyocyte morphology, and function, altered extracellular matrix composition, are the hallmarks of DCM hearts [19,20,21,22,23,24]. Such cardiac changes might result in cardiac cell damage, tissue fibrosis, ventricular dilatation, and systolic dysfunction, leading to heart failure. In line with other reports [6,9], our in silico analysis advised that the DCM involves dysregulation in angiogenesis and associated signaling pathways. Interestingly, earlier reports reveal the remedial effects of angiogenesis intervention during the DCM phenotype [12,13,14]. Therefore, more studies are needed to explore such a beneficial effect that can be utilized to develop therapeutic strategies for DCM.

Clinical studies in heart transplant patients with DCM have reported a significant decrease in cardiac proangiogenic factors (such as VEGF-A and VEGFR1) and lower blood capillary density [11], suggesting downregulation of angiogenesis during DCM phenotype. Likewise, we observed decreased angiogenic potential in DCM mice, as also supported by earlier reports in rodents [6]. Thus, these reports suggest the alternative mechanism of angiogenesis signaling in DCM pathogenesis in humans and rodents. Similarly, promoting angiogenesis during DCM has been shown to have an anti-fibrotic and cardioprotective effect [12,13,14]. Overall, this information suggests that angiogenesis could be a crucial target for DCM therapeutics. Therefore, we hypothesized that augmenting angiogenesis in the DCM heart might act as a trigger to reverse pathologic cardiac remodeling, to restore cardiac function.

Evidence from previous studies suggests that miRNAs play significant roles in cardiac remodeling [25]. Also, we have previously reported that the constitutive overexpression of miR-210 can induce cardiac angiogenesis following ischemic injury [16]. Similarly, our in vitro studies using miR-210 transfected rat cardiomyocytes culture conditioned media treated HUVEC showed a significant increase in tube formation, proposing the angiogenesis inducer properties of miR-210.

Taken together, we postulated that miR-210-mediated upregulated angiogenesis might rescue the DCM phenotype, as also advocated by earlier reports [12,13,14]. Therefore, in the present study, we used α-TM 54 mutant mice as a DCM model, which illustrates molecular, structural, and functional abnormalities similar to DCM phenotype such as myofibrillar disarray, interstitial collagen deposition indicating fibrosis, bigger hearts with dilated ventricles, and systolic and diastolic dysfunction. To evaluate the effect of angiogenesis intervention during the DCM phenotype, previously reported cardiac angiogenesis inducer miR-210 [16] was overexpressed in the DCM mice model via crossbreeding miR-210 transgenic mice (210-TG) and mutated α-TM 54 expressing DCM mice to achieve miR-210 overexpressing DCM phenotype mice (TMx210). Our results showed a significant increase in heart weight to body weight ratio in six-month-old mixed-gender TMx210 and DCM mice when compared to WT and 210-TG groups individually. Histopathological analysis, using heart tissue sections, demonstrated signs of myofibrillar loss (as indicated by myocardial disarray, and loss of cardiac tissue integrity), and interstitial fibrosis in DCM and TMx210 mice. However, by contrast with DCM, comparatively lesser interstitial fibrosis in TMx210 hearts was observed which may be due to the protective effect of miR-210 overexpression as reported earlier [16]. Also, cardiomyocyte size remained comparable in all groups, suggesting the absence of cardiac cell hypertrophy in DCM and TMx210 mice, although there were trends for bigger cell size in both of these groups. These observations suggest that a bigger cardiac size in DCM and TMx210 mice might be due to the additive effect of interstitial collagen deposition (tissue fibrosis) and uptrends in cardiomyocyte size which may also one of the reasons for cardiac dysfunction in these groups. Also, an elevated angiogenic potential was observed by ex-situ aortic culture neovascularization in TMx210 mice when compared to DCM and WT. Interestingly, there is a significant downregulation in the angiogenic potential of DCM mice aorta which is in line with previous studies in DCM patients [11]. Our in vivo study indicated an elevated cardiac blood vessel density and upregulated proangiogenic factor VEGF-A expression in TMx210 hearts as compared to DCM, suggesting improved angiogenesis in the former. Also, upregulation of VEGF, a potent angiogenic factor, in 210-TG mice heart is suggestive of miR-210 mediated VEGF pathway activation which is one of the major pathways in tissue angiogenesis.

The echocardiographic assessment showed no significant restoration of cardiac chamber dilation and heart function in TMx210 as compared to DCM. However, in contrast to DCM, there is an inhibition of LV posterior wall thinning in TMx210, indicating the partial beneficial effect of miR-210 mediated angiogenesis in TMx210 hearts.

One possible explanation for these observations may be the diverse effect of miR-210 on overall gene expression profile and associated pathways including angiogenesis signaling. In other words, miR-210-mediated upregulated angiogenesis might helpful in restoring the cardiac phenotype and physiological function in DCM mice, however, the miR-210 mediated activation of several other related genes and signaling pathways may nullify such favorable effects. Thus, more studies are needed to selectively intervene in the angiogenic signaling to examine the eventual effects during DCM development and pathogenesis.

Overall, based on these results, we conclude that miR-210-mediated upregulated angiogenesis is insufficient to rescue the DCM phenotype and cardiac function in mice.

## 5. Conclusions

Based on already reported evidence, suggesting the favorable effects of miR-210 facilitated angiogenesis during cardiac injury and associated pathogenic cardiac remodeling, the present study analyzed whether miR-210-mediated increase in cardiac angiogenesis modulates the DCM phenotype. In summary, our data confirm that miR-210 overexpression can induce upregulated cardiac angiogenesis in DCM mice; however, such an effect is not sufficient to reverse the pathologic cardiac remodeling to rescue the cardiac function.

## Data Availability

Not applicable.

## Supplementary Materials

The following are available online at https://www.mdpi.com/article/10.3390/cells10040771/s1, Figure S1: Dilated cardiomyopathy mice model. Figure S2: MiR-210 overexpressing transgenic mice model. Figure S3: Dilated cardiomyopathy mice model aging. Table S1: Echocardiographic features in mixed-gender mice at six months age. Table S2: Primer pairs for genotyping of transgenic mice. Table S3: Primer pairs for qPCR used in the current study.
