# Supplementary material for: Upregulated Angiogenesis Is Incompetent to Rescue Dilated Cardiomyopathy Phenotype in Mice
Source: Cells. 2021 Mar 31;10(4):771. doi: 10.3390/cells10040771 (PMC8066105; doi:10.3390/cells10040771)
Supplement: Supplementary file 1 [file cells-10-00771-s001.pdf]

Figure S1

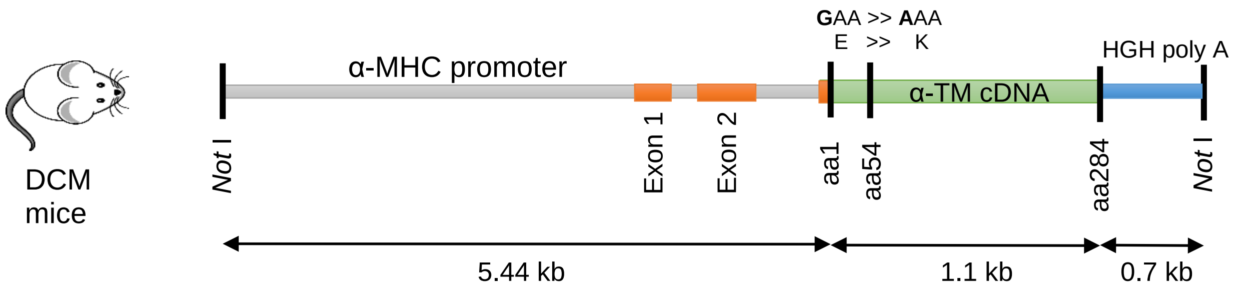

Dilated cardiomyopathy (DCM) mice model

As reported earlier,[1] DCM mutant α-TM54 (α-Tropomyosin 54) transgenic mice were generated at the University of Cincinnati Transgenic core. Briefly, the mice α-TM striated muscle-specific cDNA was cloned into the pBluescript vector. A mono-nucleotide variation (**G**AA>**A**AA) was created via site-directed mutagenesis resulting in an amino acid substitution at codon 54 (Glu54Lys). α-TM54 mutant cDNA was cloned into a vector which was contained the cardiac-specific α-MHC (α-Myosin heavy chain) promoter and the human growth hormone (HGH) poly(A) signal sequence. The transgene construct was purified to generate transgenic mice using the FVB/N strain and was identified by nucleotide sequencing of genomic DNA. Post-weaning, DNA samples were extracted from tail clips of 10 day-old pups and genotyping was done for the α-TM54 transgene using primers (Supplemental Table 2): α-MHC forward and α-TM reverse. These DCM mice demonstrate pathological and physiological phenotypes like dilated heart, ventricular dysfunction, decrease calcium sensitivity of myofilaments, brain natriuretic peptide overexpression; which are comparable to human DCM subjects.

Figure S2

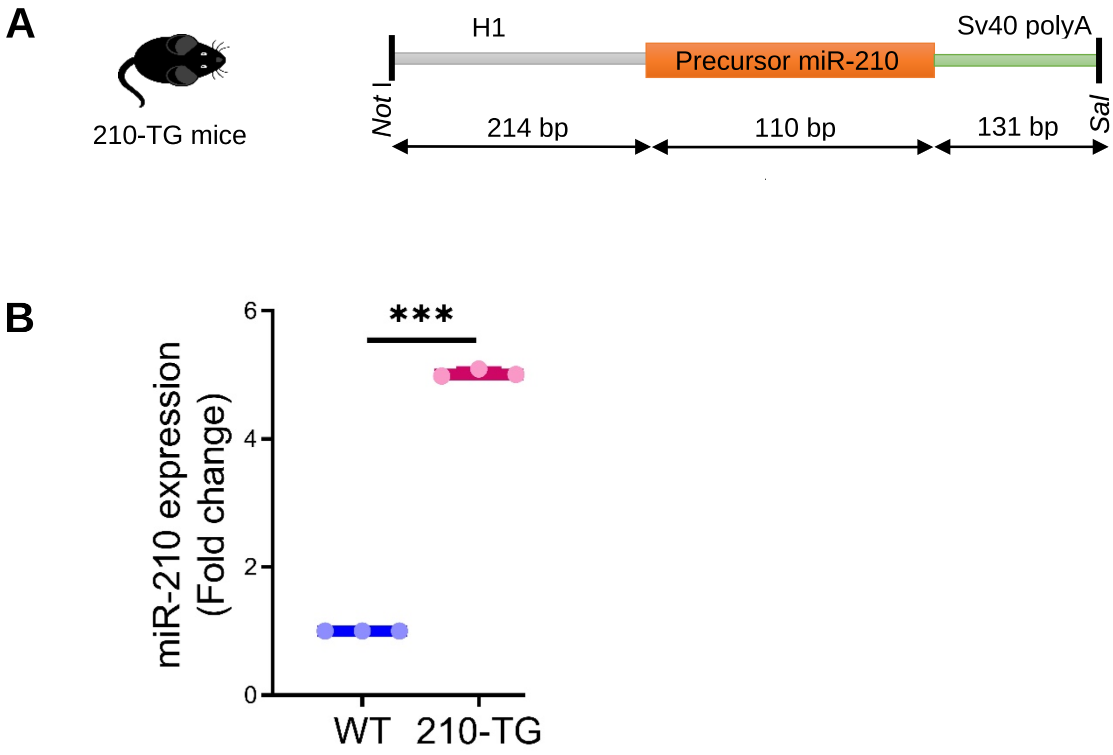

MiR-210 overexpressing transgenic mice model

A, As reported previously,[2] MiR-210 overexpressing transgenic (210-TG) mice were generated using the C57BL/6 strain. The 210-TG construct was designed to have an H1 promoter (HmiR0167-MR01 vector from GeneCopoeia, MD), precursor miR-210 cDNA, and SV40 poly A cassette at 3’ end. Precursor miR-210 along with the H1 promoter was amplified and cloned into the pShuttle vector (Stratagene, CA) using restriction enzymes Not1 and Sal1. An amplified vector was used for microinjection into the pronuclei of superovulated C57BL/6 females-derived single-cell embryos. The viable embryos were implanted into foster female mice. 210-TG and non-transgenic wild-type mice were identified by genotyping of the tail genomic DNA via PCR for custom-designed primers (Table S2).

B, Quantification of cardiac miR-210 expression by qPCR showing miR-210 overexpression in six months old 210-TG mice as compared to wild-type (WT).

Figure S3

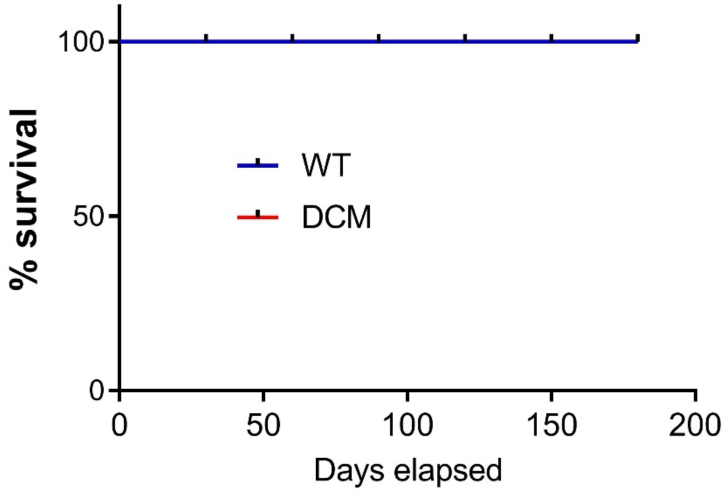

Dilated cardiomyopathy (DCM) mice model aging

Survival curve showing wild-type (WT) and DCM mice aging till six months age without any death due to heart failure.

Table S1. Echocardiographic features in mixed-gender mice at six months age

Measurement (unit)	Wild-type (n=6)	DCM (n=12)	p-value
LVEF (%)	64.2	15.3	0.002
LVFS (%)	34.6	7.0	0.002
LVID;d (mm)	3.9	5.8	0.017
LVID;s (mm)	2.5	5.5	0.005
LVPW;d (mm)	0.99	0.65	0.03
LVPW;s (mm)	1.5	0.7	0.04

Table S2. Primer pairs for genotyping of transgenic mice

Targets	Primer pairs	
	**Forward Primers**	**Reverse Primers**
miR-210	AACGCTGACGTCATCAACC	TGGTGATTTCCCAGAACACA
GAPDH	TGGCCTTCCGTGTTCCTTACC	TGTAGGCCATGAGGTCCACCAC
α-MHC	GCCCACACCAGAAATGACAGA	
α-TM		TCCAGTTCATCTTCAGTGCCC

Table S3. Primer pairs for qPCR used in the current study

Targets	Primer pairs	
	**Forward Primers**	**Reverse Primers**
miR-210	CUGUGCGUGUGACAGCGGCUGA	Universal Primer

References

1. Rajan S, Ahmed RP, Jagatheesan G, Petrashevskaya N, Boivin GP, Urboniene D, Arteaga GM, Wolska BM, Solaro RJ, Liggett SB, Wieczorek DF. Dilated cardiomyopathy mutant tropomyosin mice develop cardiac dysfunction with significantly decreased fractional shortening and myofilament calcium sensitivity. *Circ Res* 2007;101:205-214.
2. Arif M, Pandey R, Alam P, Jiang S, Sadayappan S, Paul A, Ahmed RPH. MicroRNA-210-mediated proliferation, survival, and angiogenesis promote cardiac repair post-myocardial infarction in rodents. *J Mol Med (Berl)* 2017;95:1369-1385.
